# Role of *CPI-17* in restoring skin homoeostasis in cutaneous field of cancerization: effects of topical application of a film-forming medical device containing photolyase and UV filters

**DOI:** 10.1111/exd.12177

**Published:** 2013-06-25

**Authors:** Joan Anton Puig-Butillé, Josep Malvehy, Miriam Potrony, Carles Trullas, Francisco Garcia-García, Joaquin Dopazo, Susana Puig

**Affiliations:** 1 Melanoma Unit, Biochemistry and Molecular Genetics Service, Hospital Clinic of Barcelona, IDIBAPSBarcelona, Spain; 2 CIBER de Enfermedades Raras, Instituto de Salud Carlos IIIBarcelona, Spain; 3 Melanoma Unit, Dermatology Department, Hospital Clinic of Barcelona, IDIBAPSBarcelona, Spain; 4 ISDINBarcelona, Spain; 5 Department of Bioinformatics, Centro de Investigación Príncipe FelipeValencia, Spain; 6 Functional Genomics Node, National Institute of Bioinformatics, CIPFValencia, Spain; 7 CIBER de Enfermedades Raras (CIBERER)Valencia, Spain

**Keywords:** actinic keratoses, *CPI-17*, cutaneous field of cancerization, expression array, *PPP1R14A*

## Abstract

Cutaneous field of cancerization (CFC) is caused in part by the carcinogenic effect of the cyclobutane pyrimidine dimers CPD and 6-4 photoproducts (6-4PPs). Photoreactivation is carried out by photolyases which specifically recognize and repair both photoproducts. The study evaluates the molecular effects of topical application of a film-forming medical device containing photolyase and UV filters on the precancerous field in AK from seven patients. Skin improvement after treatment was confirmed in all patients by histopathological and molecular assessment. A gene set analysis showed that skin recovery was associated with biological processes involved in tissue homoeostasis and cell maintenance. The CFC response was associated with over-expression of the *CPI-17* gene, and a dependence on the initial expression level was observed (*P* = 0.001). Low *CPI-17* levels were directly associated with pro-inflammatory genes such as *TNF* (*P* = 0.012) and *IL-1B* (*P* = 0.07). Our results suggest a role for *CPI-17* in restoring skin homoeostasis in CFC lesions.

## Background

Cutaneous field of cancerization (CFC) is associated with genomic alterations due to the carcinogenic effect of sun exposure [1] and comprises actinic keratoses (AKs) and squamous cell carcinomas (SCCs) [2]. UV radiation, particularly UVB, promotes the production of cyclobutane pyrimidine dimers (CPDs) and 6-4 photoproducts (6-4PPs) which subsequently interfere with biological processes that are critical for cell viability [3]. The nucleotide excision repair (NER) system is employed by mammal cells to remove UV-induced DNA damage [4]. However, whereas 6-4PPs are efficiently recognized and removed by the NER system, CPDs recognition and removal is poor [5,6]. Many organisms have an additional repair mechanism named photoreactivation, which is carried out by photolyases which specifically recognize and repair either CPDs or 6-4PPs [7]. The potential of DNA photolyases in skin cancer prevention has been increasingly recognized. Beneficial effects after transferring a CPD photolyase into mammals have been obtained in transgenic mice [8]. Transgenic expression of photolyases showed a 40% increase in CPD lesion repair, improved resistance against UV-induced effects suppressing the formation of skin carcinomas. Furthermore, topical application of liposome formulations with CPD photolyases onto human skin provides protection against UVB-induced damage [9].

## Questions addressed

We explored the molecular effects of topical application of Eryfotona® AK-NMSC (Eryf-AK; ISDIN, Barcelona, Spain), a film-forming medical device containing Repairsomes® (photolyase in liposomes and UV filters), in patients with CFC.

## Experimental design

For experimental design and procedures see Data S1.

## Results

Three of seven patients with CFC (AK Pretreatment biopsies) presented a complete histological clearance, one patient presented histological clearance in more than 80% of the sample, and three additional cases presented partial histological improvement associated with inflammation. Based upon the histopathology assessment after Eryf-AK treatment, the subjects were classified as fast responders (FR) versus slow-partial responders (PR) (Table S1).

**Figure 1:**
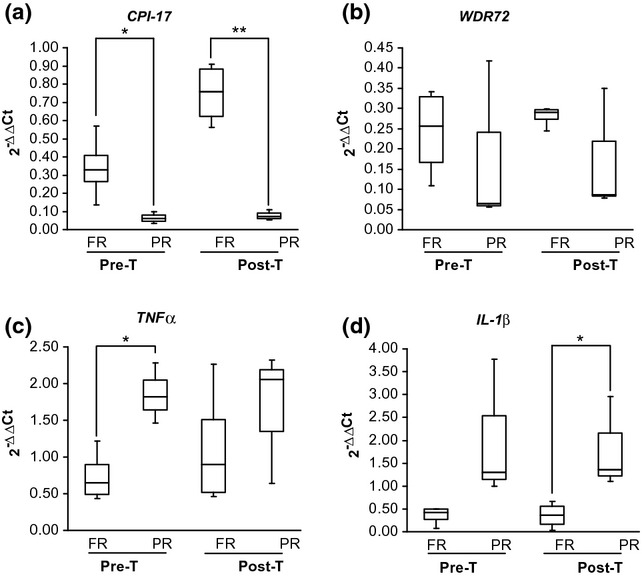
Values of relative quantification of selected genes and markers by subtype of patients (fast responders versus partial responders) and time of treatment (prior pretreatment or posttreatment): (a) Values of relative quantification of *CPI-17* (2^−ΔΔCt^). *P-values <0.05, **P-values <0.001. (b) Values of relative quantification of *WDR72* (2^−ΔΔCt^). *P*-values pretreatment lesion = 0.631; *P*-value posttreatment lesion = 0.211. (c) Values of relative quantification of *TNF*α (2^−ΔΔCt^). *P-values <0.05; *P*-value posttreatment lesion = 0.447. (d) Values of relative quantification of *IL-1B* (2^−ΔΔCt^). *P-values <0.05; *P*-value pretreatment lesion = 0.070.

The differential gene expression analysis of CFC pretreatment versus posttreatment assessment failed to detect deregulated genes after correction for multiple testing. However, over-expressions of *CPI-17* gene (2.8-fold increase, *P* = 0.039) and *WDR72* gene (1.9-fold increase, *P* = 0.040) were detected in FR subgroup. *CPI-17* expression differences between FR and PR subgroups were confirmed by RT-PCR (*P* = 0.001) (Figure 1a). In contrast, no significant differences (*P* = 0.211) were observed for *WDR72* (Figure 1b). Initial *CPI-17* levels were higher in FR than PR patients (*P* = 0.045; Figure 1a).

Pathological conditions such as inflammation modulate *CPI-17* expression [10]. Thus, expression of *TNF* and *IL-1B* cytokines were evaluated. FR showed lower expression levels of *TNF* (*P* = 0.012) and *IL-1B* (*P* = 0.07) after Eryf-AK (Figure 1c,d). Posttreatment PR lesions still showed a high *IL-1B* expression level (*P* = 0.038).

Gene set analysis identified 150 biological functions (*P* < 0.005; Table S2) associated with CFC, which were classified in four major biological clusters: ‘generation of reactive oxygen species’, ‘mechanisms involved in DNA repair’, ‘cell division process’ and ‘lipids’.

Sixty six processes were associated with Eryf-AK treatment (*P* < 0.005, Table S3), which were grouped as ‘cell communication and signalling’, ‘cell adhesion’ and ‘tissue development’.

Gene set analysis according to treatment response identified 24 GOs over-expressed in FR subgroup mostly involved in cell response and homoeostasis and 28 GO's associated with PR lesions which were related to inflammation and cytokine production, apoptosis and lipid metabolic processes (Table 1).

**Table 1:** Gene ontology terms detected by gene set analysis in posttreatment biopsies by response subgroup

Response to treatment^1^	Gene Ontology terms (ID and *P*-value)^2^
Fast responders (FR)	Cell-cell signalling (0007267, *P* = 3.02E-06); Embryonic development (0009790, *P* = 1.15E-05); Cell development (0048468, *P* = 1.46E-05); Secretion (0046903, *P* = 2.10E-05); Regulation of secretion (0051046, *P* = 1.22E-04); Muscle contraction (0006936, *P* = 1.87E-04); Chemical homoeostasis (0048878, *P* = 2.44E-04); Anterior/posterior pattern formation (0009952, *P* = 3.05E-04); Cellular homoeostasis (0019725, *P* = 4.93E-04); Embryonic development ending in birth or egg hatching (0009792, *P* = 6.73E-04); Chordate embryonic development (0043009, *P* = 1.8E-03); Homoeostatic process (0042592, *P* = 1.30E-03); Ion homoeostasis (0050801, *P* = 1.53E-03), Secretion by cell (0032940, *P* = 1,53E-03); Response to steroid hormone stimulus (0048545, *P* = 2.00E-03); Cell fate commitment (0045165, *P* = 2.19E-03); Response to hypoxia (0001666, *P* = 2.22E-03); Cellular chemical homoeostasis (0055082, *P* = 2.23E-03); Regulation of blood vessel size (0050880, *P* = 2.76E-03); Smooth muscle contraction (0006939, *P* = 3.26E-03); Response to inorganic substance (0010035, *P* = 4.09E-03); Response to hormone stimulus (0009725, *P* = 4.37E-03); Neurological system process (0050877, *P* = 3.08E-02); Cell morphogenesis (0000902, *P* = 1.17E+00)
Partial responders (PR)	Organic acid metabolic process (0006082, *P* = 3.35E-18); Carboxylic acid metabolic process (0019752, *P* = 3.35E-18); Lipid metabolic process (0006629 *P* = 1.28E-13); Cellular lipid metabolic process (0044255, *P* = 8.13E-13); Lipid biosynthetic process (0008610, *P* = 2.09E-09); Regulation of immune response (0050776, *P* = 1.09E-08); Steroid metabolic process (0008202, *P* = 7.70E-08); Regulation of interleukin-6 production (0032675, *P* = 2.07E-07); Regulation of cytokine production (0001817, *P* = 2.07E-07); Positive regulation of immune response (0050778, *P* = 2,81E-06); Carbohydrate metabolic process (0005975, *P* = 3.67E-05); Leucocyte migration (0050900, *P* = 4.62E-05); Neutral lipid metabolic process (0006638, *P* = 1.65E-04); Glycerol ether metabolic process (0006662, *P* = 1.65E-04); Regulation of lipid metabolic process (0019216, *P* = 1.80E-04); Positive regulation of cytokine production (0001819, *P* = 1.83E-04); Response to drug (0042493, *P* = 3.12E-04); cellular carbohydrate metabolic process (0044262, *P* = 3.61E-04); Locomotory behaviour (0007626, *P* = 3.91E-04); Lipid storage (0019915, *P* = 8.60E-04); Response to organic substance (0010033, *P* = 2.62E-03); Interleukin-12 production (0032615, *P* = 2.95E-03); Apoptosis (0006915, *P* = 2.96E-03); Negative regulation of cytokine production (0001818, *P* = 3.01E-03); Programmed cell death (0012501, *P* = 3.68E-03); Response to molecule of bacterial origin (0002237, *P* = 3.96E-03); Regulation of programmed cell death (0043067, *P* = 4.06E-03); Amine metabolic process (0009308, *P* = 4.74E-03)

1 Patients were classified as fast responders and partial responders based on the histopathology assessment after Eryf-AK treatment (see Table S1).

2 The ID numbers and the *P*-values are indicated for each GO.

## Conclusions

We identified biological processes associated with CFC such as ROS production and DNA damage repair processes that may be induced in part by CPDs and/or lipid metabolism. Lipid content changes are important in AK and BCC [11]. After treatment, we found over-expression of fundamental processes related to tissue reconstitution (cell communication, signalling and adhesion).

Based on treatment response, treated PR biopsies showed an over-expression of apoptotic process, lipid metabolism, cytokine production and inflammation which are directly related to AK. Inflammation is important for AK maintenance which is abolished by the topical use of diclofenac combined with hyaluronic acid through a selective inhibition of COX2 [12–14]. The histopathological evaluation showed the presence of AK in at least 20% of the biopsy specimen from PR patients. In treated FR subgroup, we observed an improvement in cell homoeostasis and adhesion which correlates with the improvement in histopathological measures (Puig et al. 2012; submitted for publication).

*CPI-17* over-expression was associated with normal phenotype recovery. *CPI-17* is one of the major Ser/Thr phosphatase isoforms, and its activation suppresses the MYPT1-PP1δ activity resulting in muscle contraction [10]. *CIP-17* expression is detected in multiple cell types [15–17] involved in several processes [16,18][. MYTP1 inhibition results in more prominent focal adhesions and absence of cell migration [19]. *CPI-17* is directly associated with focal adhesion kinases [20] and located at focal adhesions in fibroblasts and keratinocytes [21]. MYPT1-PP1δ complex can also regulate the dephosphorylation of retinoblastoma protein (pRb) [22] which shows a deregulated activation in AK. We observed that inflammation modulates *CPI-17* expression in CFC. Thus, processes such as DNA damage or ROS production may cause *CPI-17* down-deregulation, which could lead to uncontrolled MYPT1-PP1δ activity. Deregulated phosphatase activity in CFC may affect cell motility, cell adhesion and cell cycle control mediated by pRb.

In conclusion, 1-month Eryf-AK treatment improved the field of cancerization and restored normal phenotype in at least a subset of samples, through *CPI-17* up-regulation.

## Supporting Information

**Data S1.** Study design.

**Table S1.** Patient demographics and classification based on response to treatment.

**Table S2.** GO's biological processes overrepresented in CFC prior to Eryfotona AK-NMSC treatment.

**Table S3.** GO's biological processes overrepresented in lesional skin post Eryfotona AK-NMSC treatment.
