# Supplementary material for: Role of CPI-17 in restoring skin homoeostasis in cutaneous field of cancerization: effects of topical application of a film-forming medical device containing photolyase and UV filters
Source: Exp Dermatol. 2013 Jun 25;22(7):494–6. doi: 10.1111/exd.12177 (PMC3748792; doi:10.1111/exd.12177)
Supplement: Supplementary file 1 [file exd0022-0494-SD1.doc]

**Supplementary**

**DATA S1:**

**Study Design**

A pilot, prospective, controlled, interventional clinical study was performed to evaluate the effect of Eryfotona® AK-NMSC (Eryf-AK), a film-forming medical device containing Repairsomes® (DNA-repairing enzyme photolyase in liposomes and UV filters), in the treatment of the cutaneous field of cancerization (CFC).

Inclusion criteria was patients with an area larger than 3.6x3.6 cm containing multiple lesions in a sun-exposed skin area which were classified as actinic keratosis (AK) based on *in vivo* reflectance confocal microscopy assessment and later confirmed by punch biopsies.

Eleven patients with multiple scalp AK and two patients with xeroderma pigmentosum, with an area larger than 3.6x3.6 cm affected by CFC in a sun-exposed skin area, were included. Written informed consent was obtained from all patients after having read and understood the information approved by the ethics committee. The study was approved by the institutional research board and was conducted according to the Declaration of Helsinki Principles. Thirteen patients (mean age 72 years) were screened; one patient refused the treatment after the first evaluation and before the first biopsy (ery 009). Two patients refused the second biopsy due to concomitant personal issues (ery 006 and ery 011). In another three patients RNA extraction failed in one of the samples before or after treatment (ery007, ery012 and ery013).

Seven patients (two of them affected by xeroderma pigmentosum) completed the study. The test product was applied twice a day in the treatment evaluation area for four weeks, in the morning and 4-6 hours later.

**Samples**

The area assessed for CFC was divided in four 0.8x0.8 cm subareas. A 3-mm punch biopsy was obtained from area 1 for histopathological evaluation and another 3-mm punch biopsy was obtained from area 2 before starting treatment, and preserved for RNA extraction. After four weeks of Eryf-AK application, two additional punch biopsies were obtained from area 3 for histopathology exam and from area 4 for additional RNA extraction.

**RNA extraction**

Punch biopsies were embedded in RNAlater (Quiagen, US) and stored at -80ºC. Total RNA isolation was conducted using Trizol (Invitrogen Life Technologies, Carlsbad, CA) extraction-based method as indicated in the manufacturer’s protocol, followed by purification in commercial columns (Quiagen, Valencia, CA). Briefly, disruption and homogenization of skin samples was performed using Polytron System PT1200E (Kinematica, Switzerland) homogenized and lysed in Trizol. After chloroform addition, RNA was isolated from the aqueous phase. RNA was precipitated with isopropanol, washed with 70% ethanol, and redissolved in RNase-free buffer. Total isolated RNA was further purified using the RNeasy kit (Qiagen, Valencia, CA). RNA concentration was measured using a NanoDrop Spectrophotometer (Thermo Scientific) and integrity of the RNA was verified with a Bioanalyzer 2100 (Agilent, US). The RNA integrity number was higher than 7.9 for all samples.

**Expression array**

Analysis of global gene expression was performed using the Whole Human Genome (4x44k) Oligo Microarray kit (G4112F, Agilent, US). The microarray contains probes from over 41,000 specific human genes and transcripts with public domain annotations (RefSeq, Goldenpath Ensembl Unigene Human Genome and GenBank databases). Overall, 50 ng of RNA were labeled using Low input Quickamp Labeling kit (Agilent, US). In all samples, 10 commercial control probes were added to standardize the results (RNA Spike-in kit, one color, Agilent, US). The arrays were scanned using the DNA Microarray Scanner G2565CA (Agilent, US); Feature Extraction Software (Agilent, US) was used to perform the quality control process and extract the information.

**TaqMan real-time reverse transcriptase polymerase chain reaction**

To confirm the microarray results, the expression of four genes was evaluated by TaqMan real-time reverse transcriptase polymerase chain reaction (RT-PCR). cDNA was reverse-transcribed from total RNA using TaqMan PCR Core Reagment kit (Roche Applied Science, Penzbergf, Germany). RT-PCR was performed using TaqMan Universal PCR master Mix (Roche Applied Science, Penzbergf, Germany). The reaction was performed in an ABI 7900HT sequence detection instrument (Applied Biosystems, CA, US). TaqMan gene-specific primers and probes for selected genes (*CPI-17, WDR72, TNF* and *IL-1B*) were purchased from Applied Biosystems. The *GADPH* gene was used to normalize each sample. RNA from normal skin was used as a calibrator.

**Statistical analyses**

For array raw data normalization purposes, the Agilent Processed Signal was standardized across arrays using quantile normalization. Principal Component Analysis (PCA) method was used to explore the data generated by the expression arrays to assess if XP patients have a different expression that could be identified. The analyses did not identify “components” associated exclusively with the expression profile from the XP patients. Differential gene expression analysis was carried out using the Linear Models for Microarray Data (limma) package from Bioconductor (http://www.bioconductor.org). Multiple testing adjustments of P-value estimates were performed according to Benjamini and Hochberg. Gene set analysis was carried out for the Gene Ontology (GO) terms and using a logistic regression model in Babelomics (<http://babelomics.bioinfo.cipf.es/>). GO annotation for the genes in the microarray was taken from Ensembl 56 release ([http://www.ensembl.org](http://www.ensembl.org/)). Quantitative PCR of selected genes was evaluated using the relative quantification method of ΔΔCt. Expression values were evaluated by t-tests for means equality using the SPSS 17.0 software. P-values less than or equal to 0.05 were considered to be statistically significant.
