# Supplementary material for: Role of CPI-17 in restoring skin homoeostasis in cutaneous field of cancerization: effects of topical application of a film-forming medical device containing photolyase and UV filters
Source: Exp Dermatol. 2013 Jun 25;22(7):494–6. doi: 10.1111/exd.12177 (PMC3748792; doi:10.1111/exd.12177)
Supplement: Supplementary file 2 [file exd0022-0494-SD2.doc]

**Supplementary Tables:**

**Table S1: Patient demographics and classification based on response to treatment.**

XP: patient affected by xeroderma pigmentosum; M: male; F: female; AK: actinic keratosis. T0: time at patient inclusion; TF: assessment after 4-week treatment with Eryfotona AK-NMSC. ^A^ Classification based on the response after 4-week treatment (histology at TF)

| Patient | Age (y.o.) | Gender | XP | Site of lesion | Histology T0 | Histology  TF | Responders^A^ |
| --- | --- | --- | --- | --- | --- | --- | --- |
| ery001 | 92 | M | No | scalp | AK | Epidermal atrophia with focal disarranged basal layer | Fast |
| ery002 | 62 | M | No | scalp | AK | AK and lympho-eosinophilic infiltrate | Slow or partial |
| ery003 | 76 | M | No | scalp | AK | Focal changes of AK in the context of photodamaged skin | Fast |
| ary004 | 29 | F | Yes | forearm | AK | Skin with basal pigmentation and melanophages | Fast |
| ery005 | 28 | M | Yes | forearm | AK | Normal skin | Fast |
| ery008 | 79 | M | No | scalp | AK | AK and lympho-eosinophilic infiltrate | Slow or partial |
| ery010 | 67 | M | No | scalp | AK | AK | Slow or partial |

**Table S2: GO’s biological processes overrepresented in CFC prior to Eryfotona AK-NMSC treatment.**

150 GO’s were detected in the gene set analysis (levels from 3 to 9, p-value <0.005) ***).^A^*** Number of deregulated genes in the analysis. ^B^ Number of genes included in the Gene Ontology group.

| ***ID*** | ***Function*** | ***Number of genes ^A^*** | ***Total number of genes ^B^*** | ***Adjusted p value*** |
| --- | --- | --- | --- | --- |
| GO:0045184 | establishment of protein localization | 940 | 1040 | 1.37E-06 |
| GO:0015031 | protein transport | 933 | 1031 | 1.42E-06 |
| GO:0007049 | cell cycle | 850 | 954 | 7.20E-12 |
| GO:0006629 | lipid metabolic process | 873 | 949 | 3.80E-10 |
| GO:0046907 | intracellular transport | 828 | 902 | 8.60E-05 |
| GO:0044265 | cellular macromolecule catabolic process | 790 | 880 | 6.48E-04 |
| GO:0044255 | cellular lipid metabolic process | 724 | 784 | 5.39E-10 |
| GO:0006082 | organic acid metabolic process | 620 | 679 | 7.26E-14 |
| GO:0055114 | oxidation reduction | 628 | 677 | 8.81E-17 |
| GO:0005975 | carbohydrate metabolic process | 624 | 676 | 1.87E-03 |
| GO:0019752 | carboxylic acid metabolic process | 616 | 674 | 4.35E-13 |
| GO:0034613 | cellular protein localization | 605 | 659 | 9.35E-05 |
| GO:0022402 | cell cycle process | 580 | 649 | 2.65E-11 |
| GO:0006259 | DNA metabolic process | 584 | 648 | 1.13E-09 |
| GO:0051276 | chromosome organization | 525 | 637 | 1.63E-05 |
| GO:0006412 | translation | 506 | 631 | 1.81E-05 |
| GO:0033554 | cellular response to stress | 562 | 630 | 1.65E-04 |
| GO:0006886 | intracellular protein transport | 582 | 630 | 3.00E-05 |
| GO:0006396 | RNA processing | 586 | 595 | 1.07E-03 |
| GO:0006066 | alcohol metabolic process | 490 | 523 | 9.25E-05 |
| GO:0009308 | amine metabolic process | 468 | 497 | 3.83E-03 |
| GO:0046483 | heterocycle metabolic process | 472 | 484 | 4.67E-03 |
| GO:0000278 | mitotic cell cycle | 433 | 478 | 1.29E-12 |
| GO:0007608 | sensory perception of smell | 130 | 455 | 1.90E-04 |
| GO:0006519 | cellular amino acid and derivative metabolic process | 393 | 447 | 1.80E-06 |
| GO:0006974 | response to DNA damage stimulus | 382 | 429 | 1.66E-08 |
| GO:0006091 | generation of precursor metabolites and energy | 361 | 403 | 5.39E-11 |
| GO:0000279 | M phase | 341 | 394 | 1.25E-15 |
| GO:0008610 | lipid biosynthetic process | 369 | 393 | 1.92E-06 |
| GO:0034984 | cellular response to DNA damage stimulus | 353 | 389 | 9.99E-05 |
| GO:0006281 | DNA repair | 307 | 335 | 1.67E-04 |
| GO:0051726 | regulation of cell cycle | 284 | 322 | 3.82E-05 |
| GO:0006520 | cellular amino acid metabolic process | 275 | 303 | 1.05E-05 |
| GO:0051301 | cell division | 255 | 287 | 3.11E-16 |
| GO:0000087 | M phase of mitotic cell cycle | 242 | 275 | 1.53E-14 |
| GO:0006333 | chromatin assembly or disassembly | 206 | 268 | 2.12E-04 |
| GO:0007067 | Mitosis | 231 | 265 | 8.28E-15 |
| GO:0051186 | cofactor metabolic process | 236 | 261 | 8.53E-18 |
| GO:0006323 | DNA packaging | 192 | 256 | 1.28E-08 |
| GO:0008202 | steroid metabolic process | 228 | 244 | 2.80E-04 |
| GO:0006260 | DNA replication | 219 | 238 | 6.25E-15 |
| GO:0006631 | fatty acid metabolic process | 227 | 236 | 5.52E-06 |
| GO:0055085 | transmembrane transport | 195 | 222 | 1.90E-06 |
| GO:0006334 | nucleosome assembly | 161 | 222 | 1.44E-07 |
| GO:0006644 | phospholipid metabolic process | 201 | 220 | 2.84E-03 |
| GO:0016042 | lipid catabolic process | 179 | 211 | 6.94E-04 |
| GO:0006457 | protein folding | 186 | 201 | 2.78E-04 |
| GO:0015980 | energy derivation by oxidation of organic compounds | 174 | 197 | 4.18E-04 |
| GO:0006725 | cellular aromatic compound metabolic process | 178 | 193 | 1.87E-04 |
| GO:0046486 | glycerolipid metabolic process | 161 | 169 | 4.83E-03 |
| GO:0007346 | regulation of mitotic cell cycle | 140 | 166 | 1.10E-03 |
| GO:0006119 | oxidative phosphorylation | 113 | 134 | 1.22E-07 |
| GO:0022900 | electron transport chain | 110 | 132 | 2.43E-11 |
| GO:0007005 | mitochondrion organization | 115 | 129 | 1.33E-05 |
| GO:0006310 | DNA recombination | 113 | 125 | 2.02E-05 |
| GO:0042254 | ribosome biogenesis | 124 | 124 | 3.07E-04 |
| GO:0006694 | steroid biosynthetic process | 115 | 124 | 4.73E-05 |
| GO:0045333 | cellular respiration | 104 | 122 | 9.73E-10 |
| GO:0046395 | carboxylic acid catabolic process | 120 | 121 | 4.61E-06 |
| GO:0006766 | vitamin metabolic process | 106 | 120 | 4.35E-06 |
| GO:0051321 | meiotic cell cycle | 98 | 112 | 9.24E-04 |
| GO:0007126 | meiosis | 96 | 110 | 5.96E-04 |
| GO:0006518 | peptide metabolic process | 94 | 106 | 3.41E-03 |
| GO:0008654 | phospholipid biosynthetic process | 103 | 106 | 1.06E-03 |
| GO:0016125 | sterol metabolic process | 102 | 105 | 1.52E-03 |
| GO:0006633 | fatty acid biosynthetic process | 96 | 104 | 5.97E-04 |
| GO:0016072 | rRNA metabolic process | 96 | 96 | 2.10E-03 |
| GO:0042157 | lipoprotein metabolic process | 85 | 93 | 3.84E-03 |
| GO:0015992 | proton transport | 81 | 93 | 2.81E-03 |
| GO:0006364 | rRNA processing | 92 | 92 | 4.04E-03 |
| GO:0018958 | phenol metabolic process | 82 | 88 | 1.22E-03 |
| GO:0051438 | regulation of ubiquitin-protein ligase activity | 76 | 82 | 1.10E-04 |
| GO:0000075 | cell cycle checkpoint | 69 | 81 | 8.87E-06 |
| GO:0022904 | respiratory electron transport chain | 67 | 81 | 4.71E-06 |
| GO:0008033 | tRNA processing | 79 | 80 | 1.37E-04 |
| GO:0006839 | mitochondrial transport | 79 | 79 | 5.31E-06 |
| GO:0009116 | nucleoside metabolic process | 66 | 78 | 4.90E-03 |
| GO:0065002 | intracellular protein transmembrane transport | 78 | 78 | 1.02E-03 |
| GO:0051351 | positive regulation of ligase activity | 72 | 77 | 1.22E-04 |
| GO:0051443 | positive regulation of ubiquitin-protein ligase activity | 69 | 74 | 2.73E-04 |
| GO:0007059 | chromosome segregation | 69 | 73 | 6.50E-08 |
| GO:0051437 | positive regulation of ubiquitin-protein ligase activity during mitotic cell cycle | 67 | 72 | 3.36E-04 |
| GO:0051444 | negative regulation of ubiquitin-protein ligase activity | 66 | 71 | 5.42E-04 |
| GO:0042773 | ATP synthesis coupled electron transport | 57 | 71 | 1.23E-06 |
| GO:0051436 | negative regulation of ubiquitin-protein ligase activity during mitotic cell cycle | 65 | 69 | 3.24E-04 |
| GO:0043043 | peptide biosynthetic process | 62 | 67 | 1.76E-04 |
| GO:0006261 | DNA-dependent DNA replication | 60 | 67 | 3.92E-06 |
| GO:0050708 | regulation of protein secretion | 57 | 65 | 4.95E-03 |
| GO:0045454 | cell redox homeostasis | 63 | 65 | 7.86E-05 |
| GO:0006289 | nucleotide-excision repair | 52 | 63 | 2.25E-04 |
| GO:0042158 | lipoprotein biosynthetic process | 58 | 61 | 1.75E-03 |
| GO:0006308 | DNA catabolic process | 51 | 61 | 5.15E-05 |
| GO:0042775 | mitochondrial ATP synthesis coupled electron transport | 53 | 61 | 2.37E-07 |
| GO:0006302 | double-strand break repair | 56 | 59 | 5.52E-06 |
| GO:0006354 | RNA elongation | 51 | 57 | 3.48E-03 |
| GO:0006497 | protein amino acid lipidation | 54 | 56 | 7.15E-04 |
| GO:0019395 | fatty acid oxidation | 52 | 52 | 1.91E-06 |
| GO:0009451 | RNA modification | 50 | 50 | 1.54E-03 |
| GO:0007051 | spindle organization | 39 | 50 | 3.11E-03 |
| GO:0007093 | mitotic cell cycle checkpoint | 40 | 48 | 3.21E-04 |
| GO:0009239 | enterobactin biosynthetic process | 41 | 48 | 1.29E-05 |
| GO:0006220 | pyrimidine nucleotide metabolic process | 41 | 42 | 2.88E-04 |
| GO:0016126 | sterol biosynthetic process | 42 | 42 | 1.33E-04 |
| GO:0009062 | fatty acid catabolic process | 39 | 41 | 3.99E-05 |
| GO:0009060 | aerobic respiration | 38 | 40 | 3.04E-06 |
| GO:0006071 | glycerol metabolic process | 37 | 37 | 4.58E-03 |
| GO:0006505 | GPI anchor metabolic process | 36 | 36 | 2.61E-03 |
| GO:0006084 | acetyl-CoA metabolic process | 30 | 33 | 1.22E-03 |
| GO:0006695 | cholesterol biosynthetic process | 32 | 33 | 7.92E-05 |
| GO:0000819 | sister chromatid segregation | 30 | 33 | 1.03E-04 |
| GO:0007006 | mitochondrial membrane organization | 32 | 32 | 3.44E-04 |
| GO:0000070 | mitotic sister chromatid segregation | 29 | 32 | 1.60E-04 |
| GO:0006626 | protein targeting to mitochondrion | 26 | 29 | 1.25E-06 |
| GO:0009262 | deoxyribonucleotide metabolic process | 27 | 28 | 2.02E-04 |
| GO:0006284 | base-excision repair | 27 | 27 | 3.66E-03 |
| GO:0030262 | apoptotic nuclear changes | 21 | 25 | 4.96E-03 |
| GO:0006270 | DNA replication initiation | 22 | 25 | 6.82E-06 |
| GO:0007031 | peroxisome organization | 23 | 23 | 1.12E-04 |
| GO:0050709 | negative regulation of protein secretion | 14 | 22 | 3.18E-03 |
| GO:0007098 | centrosome cycle | 20 | 22 | 4.22E-04 |
| GO:0008299 | isoprenoid biosynthetic process | 18 | 21 | 2.96E-06 |
| GO:0048535 | lymph node development | 9 | 20 | 2.75E-03 |
| GO:0065005 | protein-lipid complex assembly | 13 | 19 | 1.21E-03 |
| GO:0000038 | very-long-chain fatty acid metabolic process | 16 | 18 | 2.25E-03 |
| GO:0006309 | DNA fragmentation involved in apoptosis | 13 | 18 | 2.49E-03 |
| GO:0000724 | double-strand break repair via homologous recombination | 18 | 18 | 1.21E-07 |
| GO:0006297 | nucleotide-excision repair, DNA gap filling | 16 | 17 | 4.71E-04 |
| GO:0006637 | acyl-CoA metabolic process | 17 | 17 | 3.24E-04 |
| GO:0008272 | sulfate transport | 15 | 16 | 3.22E-03 |
| GO:0008209 | androgen metabolic process | 10 | 16 | 1.50E-03 |
| GO:0019363 | pyridine nucleotide biosynthetic process | 15 | 15 | 3.84E-04 |
| GO:0009264 | deoxyribonucleotide catabolic process | 14 | 14 | 2.86E-03 |
| GO:0034384 | high-density lipoprotein particle clearance | 8 | 14 | 1.74E-03 |
| GO:0006625 | protein targeting to peroxisome | 13 | 13 | 4.39E-03 |
| GO:0007625 | grooming behavior | 9 | 12 | 3.95E-03 |
| GO:0042375 | quinone cofactor metabolic process | 12 | 12 | 6.01E-04 |
| GO:0051298 | centrosome duplication | 9 | 12 | 1.51E-04 |
| GO:0033108 | mitochondrial respiratory chain complex assembly | 11 | 11 | 1.87E-03 |
| GO:0031649 | heat generation | 7 | 10 | 4.44E-03 |
| GO:0006312 | mitotic recombination | 9 | 9 | 1.83E-03 |
| GO:0007007 | inner mitochondrial membrane organization | 9 | 9 | 7.21E-04 |
| GO:0009263 | deoxyribonucleotide biosynthetic process | 7 | 8 | 9.61E-05 |
| GO:0022616 | DNA strand elongation | 7 | 7 | 2.90E-03 |
| GO:0060192 | negative regulation of lipase activity | 7 | 7 | 1.21E-04 |
| GO:0034379 | very-low-density lipoprotein particle assembly | 7 | 7 | 9.44E-05 |
| GO:0042769 | DNA damage response, detection of DNA damage | 6 | 6 | 4.36E-03 |
| GO:0000089 | mitotic metaphase | 5 | 6 | 3.33E-03 |
| GO:0015802 | basic amino acid transport | 5 | 6 | 1.37E-03 |
| GO:0006271 | DNA strand elongation during DNA replication | 5 | 5 | 5.30E-04 |
| GO:0051005 | negative regulation of lipoprotein lipase activity | 5 | 5 | 5.19E-04 |

**Table S3: GO’s biological processes overrepresented in lesional skin post Eryfotona AK-NMSC treatment.**

66 GO’s were identified in the gene set analysis (levels from 3 to 9, p-value <0.005). ^A^ Number of deregulated genes in the analysis. ^B^ Number of genes included in the Gene Ontology group.

| ***ID*** | ***Function*** | ***Number of genes ^A^*** | ***Total number of genes ^B^*** | ***Adjusted p value*** |
| --- | --- | --- | --- | --- |
| GO:0007399 | nervous system development | 965 | 1136 | 2.09E-04 |
| GO:0010646 | regulation of cell communication | 957 | 1070 | 5.03E-05 |
| GO:0007155 | cell adhesion | 888 | 953 | 3.77E-05 |
| GO:0048468 | cell development | 782 | 921 | 7.65E-04 |
| GO:0009966 | regulation of signal transduction | 824 | 912 | 1.54E-05 |
| GO:0006468 | protein amino acid phosphorylation | 893 | 893 | 2.54E-05 |
| GO:0009888 | tissue development | 699 | 808 | 5.10E-05 |
| GO:0009887 | organ morphogenesis | 711 | 800 | 3.26E-04 |
| GO:0006928 | cellular component movement | 588 | 666 | 5.96E-04 |
| GO:0009790 | embryonic development | 537 | 619 | 5.63E-04 |
| GO:0010629 | negative regulation of gene expression | 439 | 499 | 4.62E-03 |
| GO:0048870 | cell motility | 424 | 484 | 1.67E-03 |
| GO:0016481 | negative regulation of transcription | 398 | 458 | 3.37E-03 |
| GO:0007167 | enzyme linked receptor protein signaling pathway | 426 | 432 | 1.51E-04 |
| GO:0016477 | cell migration | 350 | 405 | 1.58E-03 |
| GO:0001501 | skeletal system development | 374 | 394 | 1.65E-03 |
| GO:0048871 | multicellular organismal homeostasis | 291 | 339 | 1.31E-03 |
| GO:0048598 | embryonic morphogenesis | 287 | 334 | 7.41E-04 |
| GO:0007265 | Ras protein signal transduction | 309 | 316 | 9.15E-05 |
| GO:0030029 | actin filament-based process | 273 | 299 | 4.18E-03 |
| GO:0001944 | vasculature development | 277 | 294 | 1.86E-04 |
| GO:0001568 | blood vessel development | 272 | 288 | 1.67E-04 |
| GO:0007169 | transmembrane receptor protein tyrosine kinase signaling pathway | 269 | 276 | 2.18E-03 |
| GO:0051056 | regulation of small GTPase mediated signal transduction | 270 | 270 | 8.21E-06 |
| GO:0009409 | response to cold | 217 | 266 | 3.02E-03 |
| GO:0006816 | calcium ion transport | 226 | 248 | 4.20E-03 |
| GO:0008015 | blood circulation | 225 | 247 | 1.05E-03 |
| GO:0042309 | homoiothermy | 198 | 244 | 2.18E-03 |
| GO:0050826 | response to freezing | 197 | 243 | 2.24E-03 |
| GO:0007507 | heart development | 202 | 230 | 4.65E-04 |
| GO:0046578 | regulation of Ras protein signal transduction | 221 | 221 | 4.33E-05 |
| GO:0048729 | tissue morphogenesis | 168 | 193 | 9.58E-04 |
| GO:0031589 | cell-substrate adhesion | 152 | 168 | 7.48E-06 |
| GO:0006814 | sodium ion transport | 145 | 155 | 1.28E-03 |
| GO:0007178 | transmembrane receptor protein serine/threonine kinase signaling pathway | 142 | 145 | 2.66E-04 |
| GO:0009187 | cyclic nucleotide metabolic process | 121 | 141 | 8.94E-04 |
| GO:0030155 | regulation of cell adhesion | 123 | 140 | 1.28E-03 |
| GO:0007160 | cell-matrix adhesion | 125 | 138 | 3.53E-04 |
| GO:0060173 | limb development | 98 | 113 | 1.70E-04 |
| GO:0030198 | extracellular matrix organization | 96 | 110 | 1.97E-06 |
| GO:0035108 | limb morphogenesis | 95 | 109 | 2.12E-04 |
| GO:0030326 | embryonic limb morphogenesis | 86 | 96 | 8.22E-04 |
| GO:0007179 | transforming growth factor beta receptor signaling pathway | 83 | 89 | 1.30E-03 |
| GO:0002573 | myeloid leukocyte differentiation | 59 | 77 | 1.03E-03 |
| GO:0003007 | heart morphogenesis | 73 | 77 | 1.24E-03 |
| GO:0048771 | tissue remodeling | 69 | 76 | 4.11E-04 |
| GO:0045785 | positive regulation of cell adhesion | 57 | 68 | 7.96E-04 |
| GO:0030509 | BMP signaling pathway | 60 | 61 | 1.31E-03 |
| GO:0002455 | humoral immune response mediated by circulating immunoglobulin | 35 | 44 | 3.32E-03 |
| GO:0046849 | bone remodeling | 33 | 36 | 3.05E-03 |
| GO:0007602 | phototransduction | 32 | 35 | 1.21E-03 |
| GO:0010811 | positive regulation of cell-substrate adhesion | 31 | 32 | 9.77E-04 |
| GO:0045216 | cell-cell junction organization | 30 | 30 | 3.98E-03 |
| GO:0030199 | collagen fibril organization | 28 | 28 | 2.37E-04 |
| GO:0030048 | actin filament-based movement | 21 | 26 | 3.32E-04 |
| GO:0034103 | regulation of tissue remodeling | 20 | 20 | 4.12E-03 |
| GO:0035116 | embryonic hindlimb morphogenesis | 17 | 18 | 2.64E-03 |
| GO:0030901 | midbrain development | 15 | 17 | 3.79E-03 |
| GO:0043462 | regulation of ATPase activity | 11 | 15 | 5.77E-04 |
| GO:0032964 | collagen biosynthetic process | 14 | 15 | 1.18E-04 |
| GO:0060070 | Wnt receptor signaling pathway through beta-catenin | 13 | 15 | 4.07E-03 |
| GO:0014910 | regulation of smooth muscle cell migration | 12 | 12 | 3.79E-03 |
| GO:0007168 | receptor guanylyl cyclase signaling pathway | 6 | 6 | 5.99E-05 |
| GO:0043171 | peptide catabolic process | 6 | 6 | 1.50E-04 |
| GO:0001957 | intramembranous ossification | 5 | 5 | 3.23E-03 |
| GO:0010815 | bradykinin catabolic process | 2 | 2 | 2.30E-04 |
